# Identification and Validation of Immune-Related Biomarker Gene and Construction of ceRNA Networks in Septic Cardiomyopathy

**DOI:** 10.3389/fcimb.2022.912492

**Published:** 2022-06-16

**Authors:** Jingru Li, Xinyu Wu, Chaozhong Li, Guihu Sun, Peng Ding, Yanyan Li, Ping Yang, Min Zhang, Luqiao Wang

**Affiliations:** ^1^ Department of Cardiology, The First Affiliated Hospital of Kunming Medical University, Kunming, China; ^2^ Department of Emergency, The First Affiliated Hospital of Kunming Medical University, Kunming, China

**Keywords:** septic cardiomyopathy, non-coding RNA, immune-related gene, biomarker, ceRNA network

## Abstract

Septic cardiomyopathy (SCM) is a cardiac dysfunction caused by severe sepsis, which greatly increases the risk of heart failure and death, and its molecular mechanism is unclear. The immune response has been reported to be an important process in septic cardiomyopathy and is present in the cardiac tissue of patients with sepsis, suggesting that the immune response may be an underlying mechanism of myocardial injury in SCM. Therefore, we explored the role of immune-related genes (IRGs) in SCM and aimed to identify pivotal immune-related targets with the aim of identifying key immune-related targets in SCM and potential therapeutic mechanisms involved in the pathological process of SCM. To explore the regulatory mechanisms of immune responses in SCM, we identified differentially expressed genes (DEGs) shared in the SCM datasets GSE179554 and GSE40180 by bioinformatics analysis and then obtained hub genes from the DEGs. Then, we obtained the immune-related hub genes (IRHGs) by intersecting the hub genes with IRGs and performed quantitative reverse transcription polymerase chain reaction to confirm the abnormal expression of IRHGs. Finally, we further constructed an immune-related lncRNA–miRNA–IRHG ceRNA regulatory network. In this study, we identified an IRHG that may be involved in the pathogenesis of SCM, which helps us to further elucidate the role of immune response in SCM and gain insights into the molecular mechanisms and potential therapeutic targets of SCM.

## Introduction

Septic cardiomyopathy (SCM) is a systemic, multi-organ–linked malignant disease induced by sepsis, which often leads to host immune system disorders, organ dysfunction, and even death (Beesley et al., 2018). Not only that, but when patients with sepsis develop myocardial dysfunction, the mortality rate increases from 10% to 40% (Singer et al., 2016). At present, the diagnosis of SCM is still mainly based on echocardiography (Annane et al., 2005) and myocardial injury markers (Charpentier et al., 2004; Romero-Bermejo et al., 2011), and early intervention for SCM cannot be performed. Therefore, the exploration of novel biomarkers for early diagnosis and intervention of SCM may be crucial to reducing the mortality of patients with SCM.

Non-coding RNAs (ncRNAs) [including long ncRNAs (lncRNAs) and microRNAs (miRNAs)] are transcripts with a variety of biological regulatory functions and are key regulators of many biological processes (BPs) such as proliferation, differentiation, apoptosis, and metabolism. Furthermore, competing endogenous RNA (ceRNA) networks, composed of lncRNAs that share miRNA response elements (MREs) with mRNAs, play regulatory roles in a variety of diseases, including sepsis (An et al., 2021) and SCM (Pei et al., 2021). Pei et al. revealed that the lncRNA TTN-AS1/miR-29a/E2F2 ceRNA network could successfully reduce SCM-induced myocardial injury (Pei et al., 2021). Therefore, the ceRNA network established by ncRNAs and mRNAs is likely to be a key regulatory mechanism in the progression of SCM.

The immune response is the body’s defense against external damage. In septic cardiomyopathy caused by sepsis, immune response is an important pathological process. Current research on immune responses in SCM has focused on the role of pro-inflammatory mediators at the molecular and cellular levels (Hoesel et al., 2007). For example, Zhang et al. found that inhibition of TLR4/NF-kB/TNF-a/IL-18 immune signaling in cardiomyocytes could prevent LPS-induced cardiac dysfunction in mice (Zhang et al., 2020). It is suggested that immune regulation may be an important part of myocardial injury in SCM. However, the exact mechanism of immune response in sepsis-induced myocardial injury remains unclear.

**Figure 1:**
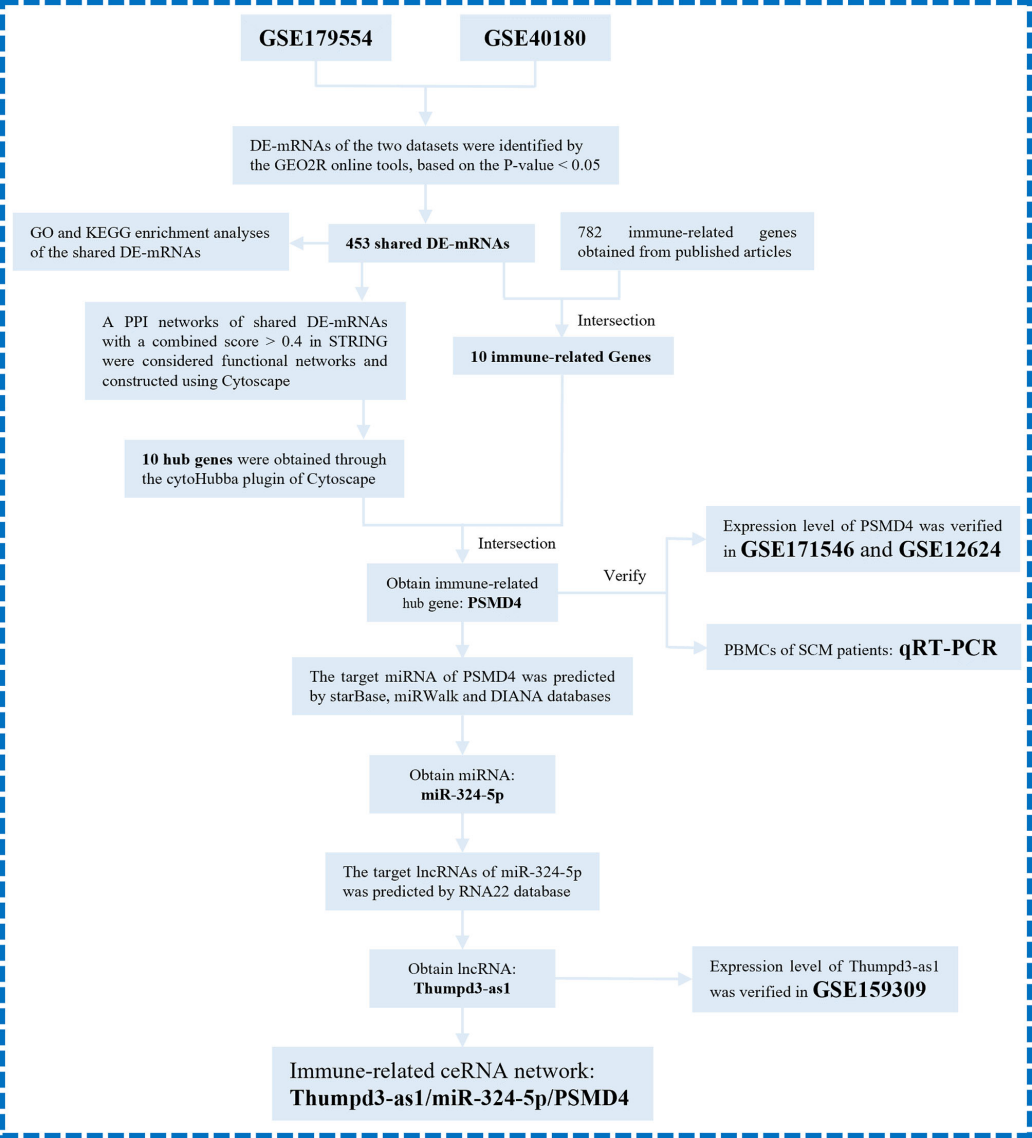
The overall protocol of this study. DE-mRNAs, differentially expressed mRNAs; GO, Gene Ontology; KEGG, Kyoto Encyclopedia Genes and Genomes; PPI, protein–protein interaction.

Here, to determine the role of immune responses in SCM, we obtained differentially expressed genes (DEGs) shared in datasets GSE179554 and GSE40180 by bioinformatics analysis. Gene Ontology (GO) and Kyoto Encyclopedia of Genes and Genomes (KEGG) pathway enrichment analysis of shared DEGs were performed subsequently. On the basis of the STRING online database, we established a protein–protein interaction (PPI) network of shared DEGs and used the cytoHubba plugin to screen out hub genes from the PPI network. We then analyzed shared DEGs and immune-related genes (IRFs) to obtain IRGs in SCM. The IRHGs in SCM were finally obtained after the intersection of Hub gene and IRGs and verified by quantitative reverse transcription polymerase chain reaction (qRT-PCR) and dataset to confirm the abnormal expression of IRHGs. Not only that, after screening the target IRHG, we obtained the upstream miRNAs and lncRNAs of IRHG from the relevant database. Then, key immune-related lncRNA–miRNA–IRHG ceRNA networks were constructed after dataset validation. We summarize the overall flow chart of this study and present it in **Figure 1**.

## Materials and Methods

### Expression Profile of mRNA and lncRNA in Microarray Data From Mice and Rat With Septic Cardiomyopathy

Five septic cardiomyopathy mouse, rat, and human datasets (GSE179554, GSE40180, GSE171546, GSE159309 and GSE12624) were obtained from the National Institutes of Health (NIH)–National Center for Biotechnology Information (NCBI)–Gene Expression Omnibus (GEO) database (https://www.ncbi.nlm.nih.gov/gds/). Details of these GEO datasets are presented in **Table 1**. Myocardial tissues from septic cardiomyopathy mice were used in all five databases, and expression profiles were analyzed by high-throughput sequencing (GSE179554 and GSE171546) and array sequencing (GSE40180, GSE159309, and GSE12624), respectively. Therefore, we used these five datasets for the acquisition of SCM immune-related biomarker genes and the construction of ceRNA networks. It is important to note that our technique is achievable and accepted, and gene expression under pathophysiological conditions has been studied by scholars (Xu et al., 2020; Cheng et al., 2021).

**Table 1:** Information of selected microaray datasets.

GEO Accession	Experiment Type	Species	Experimental Model	Source Tissue	Sample		Data	Attribute
					Control	SCM		
GSE179554	High-throughput sequencing	Mice	CLP-induced	Myocardial tissue	4	4	mRNA	Test set
GSE40180	Array	Mice	CLP-induced	Myocardial tissue	5	5	mRNA	Test set
GSE171546	High-throughput sequencing	Mice	CLP-induced	Myocardial tissue	5	5	mRNA	Validation set
GSE159309	Array	Rat	LPS-induced	Myocardial tissue	5	5	lncRNA	Validation set
GSE12624	Array	Human	Multiple Trauma	Peripheral whole blood	36	34	mRNA	Validation set

CLP, cecal ligation and puncture; LPS, lipopolysaccharide.

### Functional Enrichment Analyses

The biological functions of recognized DE-mRNAs of pastime had been assessed through the usage of the Metascape bioinformatics sources (Metascape, a gene annotation and analysis resource, http://metascape.org, version 3.5) (Zhou et al., 2019). DE-mRNAs had been first screened from the dataset, and then, the shared DE-mRNAs have been imported into Metascape for GO and KEGG enrichment analysis. For GO analyses, enriched BPs, molecular functions (MFs), and cellular components (CCs) have been assessed.

### Protein–Protein Interaction Analysis

PPI networks have been generated from the STRING database (https://string-db.org/) (Szklarczyk et al., 2021). A rating of 0.4 (medium confidence) used to be set as a threshold. Next, to obtain permission from the central gene, we imported the PPI evaluation consequences into the Cytoscape software program software (version 3.4.0, https://cytoscape.org/) (Shannon et al., 2003), using the cytoHubba plugin (Chin et al., 2014) perceived vital genes in this neighborhood as hub genes. We used five algorithms, specifically Degree, Maximal Clique Centrality (MCC), Maximum Neighborhood Component (MNC), Density of MNC (DMNC), and Clustering Coefficient, to calculate the top 10 hub genes.

### Identification of Immune-Related Hub Genes

To also elucidate whether the shared DEGs have immune-related functions, we obtained 782 immune cell marker genes from a previously published article (Bindea et al., 2013) and intersected the shared DEGs with immune cell marker genes to obtain IRGs in SCMs. DEGs that overlap with immune cell-specific marker genes are defined as immune-associated DEGs. Then, we crossed the top 10 hub genes with IRGs to obtain immune-related hub genes (IRHGs) in SCM. For detailed research methods, refer to the published articles (Yu et al., 2021).

### SCM Sample Collection and Quantitative Reverse Transcription Polymerase Chain Reaction Analysis

Peripheral blood mononuclear cell (PBMC) samples of patients with septic cardiomyopathy were collected from the Department of Emergency Medicine of the First Affiliated Hospital of Kunming Medical University, Yunnan Province, and medical information was collected in accordance with the Declaration of Helsinki after informed consent of the patients. This study was approved by the Ethics Committee of the First Affiliated Hospital of Kunming Medical University, Yunnan Province (2022) ethical review L, No.23). Patients with a clear history of sepsis and reduced left ventricular ejection fraction (LVEF) <0.55 were included in the study. Total RNA was isolated using TRIzol reagent (Invitrogen) followed by amplification grade (Invitrogen) DNase 1 for further processing. TaqMan Reverse Transcription Reagents (Applied Biosystems) were used for cDNA synthesis. qRT-PCR was performed in a My iQ Thermal Cycler (Bio-Rad) using 2× iQ SYBR Green Supermix (Bio-Rad). Each reaction contains 1 µl of cDNA for a total volume of 20 µl. Using glyceraldehyde-3-phosphate dehydrogenase (GAPDH) as the internal control of the target gene, the 2^−ΔCt^ method was used for data analysis. PCR primers were designed for human sequences and synthesized by Invitrogen. The primer sequences for PSMD4 are as follows: forward: 5′-CAACGTGGGCCTTATCACAC-3′ and reverse: 5′-ATTGTCCTCCACTGGGCTTC-3′. GAPDH primer sequences are as follows: forward: 5′-GGCCTCCAAGGAGTAAGACC-3′ and reverse: 5′-AGGGGAGATTCAGTGTGGTG-3′. The appearance of a single-peak in the melt-curve suggested the specificity of the PCR products.

### Prediction of Target miRNAs

We used three online miRNA databases, namely, starBase v2.0 (http://starbase.sysu.edu.cn/) (Li et al., 2014), miRWalk (http://mirwalk.umm.uni-heidelberg.de/) (Sticht et al., 2018), and DIANA-TarBase (http://www.microrna.gr/tarbase) (Karagkouni et al., 2018), to predict the target miRNA of the target gene. The database prediction results of target miRNAs of selected mRNAs are displayed using a Venn diagram.

### Construction of ceRNA Networks

RNA22 (https://cm.jefferson.edu/rna22/Interactive/) (Loher and Rigoutsos, 2012) was used to predict lncRNAs that interacted with the selected miRNAs. The predicted lncRNAs of were intersected with the GSE159309-lncRNA database to obtain the intersection lncRNA. We selected the lncRNA with the largest fold change and entered NCBI as the target-lncRNA. Then, the ceRNA network based on the interaction between mRNA, miRNA, and lncRNA was constructed using Cytoscape.

### Statistical Analysis of Microarray Data

The two-tailed Students’ t-test was once used to pick out the variations between groups. We used the R statistical evaluation bundle (version 3.5.3) to statistically analyze the data. An alpha price of P < 0.05 was once measured and regarded as a statistically tremendous match or evaluation in this study.

## Result

### Identification of DE-mRNAs in Myocardial Tissue of SCM Mice and Rat

**Figure 2:**
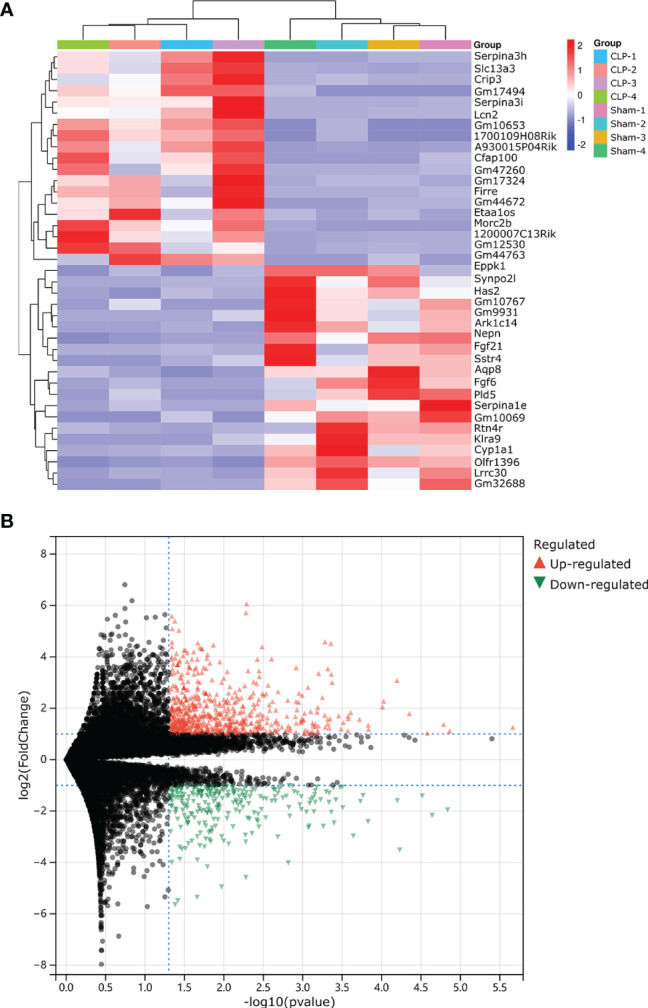
Detection of differentially expressed mRNAs (DE-mRNAs) of mice myocardial tissue in the GSE179554 dataset. **(A)** An expression heatmap corresponding to the expression profile of mRNAs in the mice myocardial tissue. **(B)** A volcano plot corresponding to the expression profile of mRNAs in the mice myocardial tissue. The red plots represent upregulated genes, the black plots represent nonsignificant genes, and the green plots represent downregulated genes.

**Figure 3:**
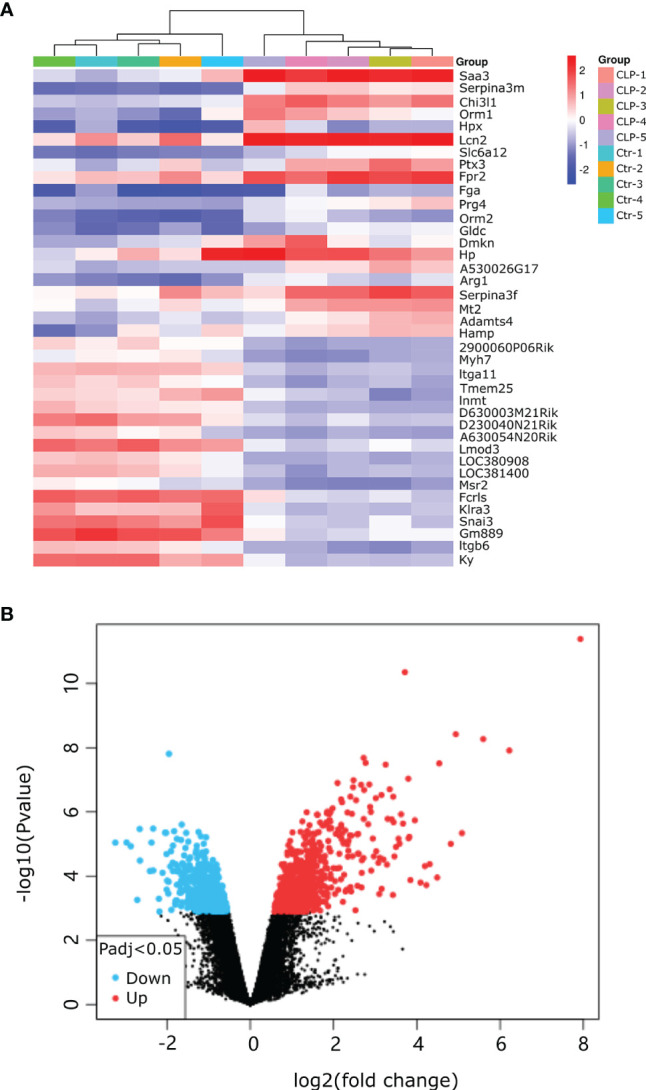
Detection of differentially expressed mRNAs (DE-mRNAs) of mice myocardial tissue in the GSE40180 dataset. **(A)** An expression heatmap corresponding to the expression profile of mRNAs in the mice myocardial tissue. **(B)** A volcano plot corresponding to the expression profile of mRNAs in the mice myocardial tissue. The red plots represent upregulated genes, the black plots represent nonsignificant genes, and the blue plots represent downregulated genes.

Our research covers GEO datasets GSE179554 and GSE40180. GSE179554 has a total of 104 samples, from which we selected four normal myocardial tissue data as the control group and four cecal ligation and puncture (CLP) mouse model myocardial tissue data as the test group for analysis. GSE40180 has a total of 72 samples, from which we extracted five normal control myocardial tissue data and five CLP mouse model myocardial tissue data for subsequent analysis. The cluster heatmap and volcano plot in **Figures 2A, B**, **3A, B** show the data distribution of the GSE179554 and GSE40180 datasets, respectively. In the GSE179554 dataset, a total of 2,198 differentially expressed mRNAs (DEGs, *P* < 0.05) were identified (1,665 upregulated and 533 downregulated). In the GSE40180 dataset, a total of 5,720 differentially expressed mRNAs (DEGs, *P* < 0.05) were identified (2,578 upregulated and 3,142 downregulated). Then, we crossed the differential genes of GSE179954 and GSE40180 to obtain 453 shared DEGs and used these differential genes for subsequent analysis.

### Pathway Enrichment Analyses

**Figure 4:**
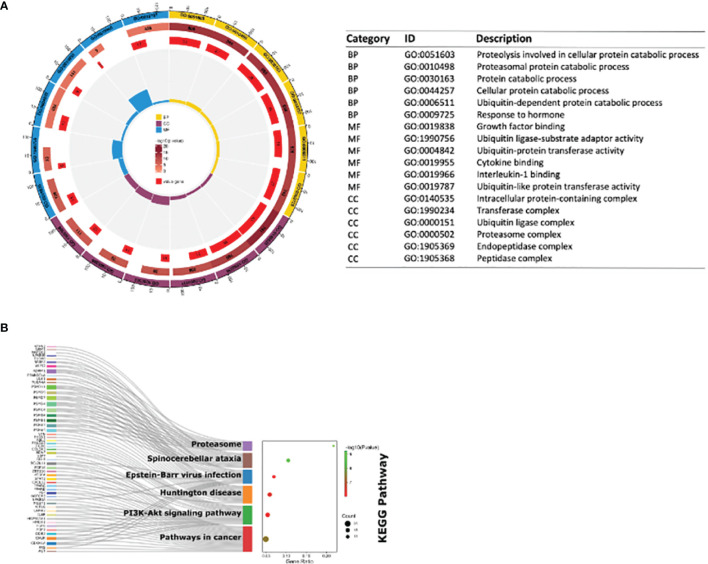
GO enrichment and KEGG pathway of shared DE-mRNAs in mouse myocardial tissues in GSE179554 and GSE40180 datasets. **(A)** GO term enrichment analysis results. The circle diagram shows the top 6 GO enriched terms in BP, MF, and CC. GO term enrichment analysis results. The circle diagram is from outside to inside: the outermost circle represents the term of GO; the second circle represents the total number of enriched genes in that term; the darker the color, the smaller the P-value of the entry; the third circle represents the number of overlaps between our input genes and the total genes of the entry; the circular histogram in the middle represents the enrichment ratio of the input gene. The higher the column, the more obvious the enrichment degree. **(B)** The bubble plot shows the most enriched KEGG pathways of DE-mRNAs. The most significant KEGG pathways are involved in proteasome, spinocerebellar ataxia and Epstein–Barr virus infection pathway. GO, Gene Ontology; BP, biological process; MF, molecular function; CC, cellular components; KEGG, Kyoto Encyclopedia Genes and Genomes; the screening criteria for significant enriched biological processes and pathways were Q < 0.05. The Q-value is the adjusted p-value.

The consequences of GO analysis indicated that these shared DEGs were associated with diverse BPs, MFs, and CCs. The top three BPs those shared DEGs were involved in were proteasomal protein catabolic process, proteolysis involved in cellular protein catabolic process, and ubiquitin-dependent protein catabolic process; the top three significant MFs related to those shared DEGs were interleukin-1 binding, ubiquitin ligase-substrate adaptor activity, and growth factor binding; the top three significant CCs in which these shared DEGs participated were proteasome complex, endopeptidase complex, and peptidase complex (**Figure 4A**). KEGG pathway analysis showed those DE-mRNAs to be significantly enriched in proteasome, spinocerebellar ataxia, and Epstein–Barr virus infection pathway (**Figure 4B**).

### PPI Network Analysis and CytoHubba Gene Identification

**Figure 5:**
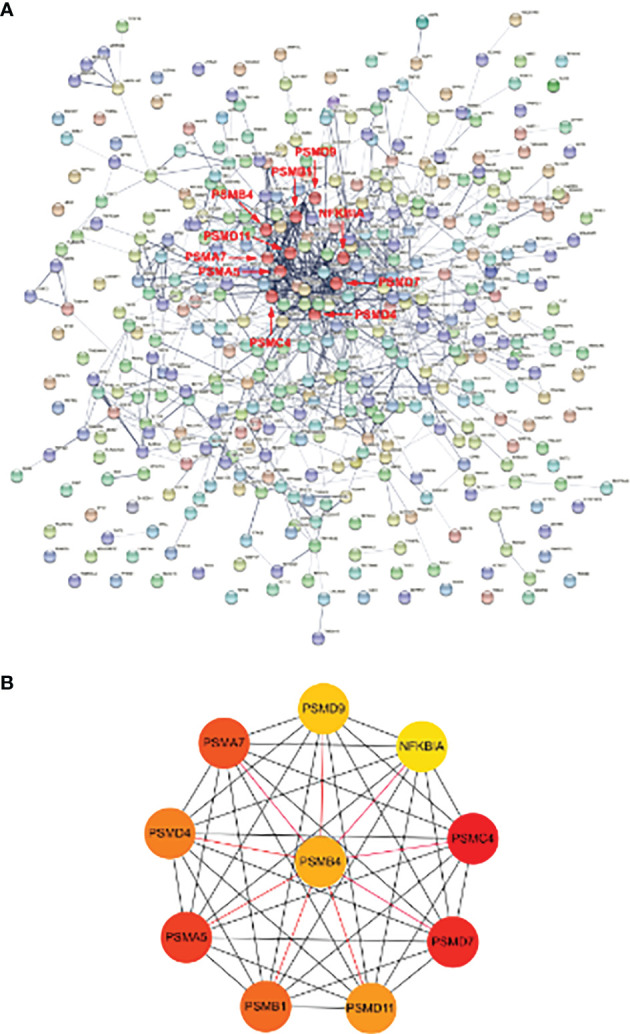
PPI network of DE-mRNAs and hub genes identification. **(A)** The interaction network between proteins coded by DE-mRNAs was composed of 417 nodes and 922 edges. Each node represents a protein, whereas each edge represents one protein–protein association. The smaller the value of Q, the larger the shape size. **(B)** Cluster plots represent the top 10 hub genes identified by cytoHubba. Associations of hub genes are marked with red markers in the PPI network.

The interaction network between proteins coded by shared DEGs, which was composed of 417 nodes and 922 edges, was constructed and visualized by STRING (**Figure 5A**). Next, we used the cytoHubba plugin to identify hub genes. The top 10 hub genes (**Figure 5B**) were identified by intersecting the results from the five algorithms of cytoHubba including Degree, MCC, MNC, DMNC, and Clustering Coefficient. Finally, we got the top 10 hub genes: PSMC4, PSMD7, PSMA5, PSMA7, PSMB1, PSMD4, PSMD11, PSMB4, PSMD9, and NFKBIA. Details of these hub genes are shown in **Table 2**. These genes are the most important in the PPI network and may play an important role in the pathogenesis of SCM.

**Table 2:** Top 10 hub genes identified by five algorithms of cytoHubba.

Gene symbol	Description
PSMC4	Proteasome 26S subunit, ATPase 4
PSMD7	Proteasome 26S subunit, non–ATPase 7
PSMA5	Proteasome 20S subunit alpha 5
PSMA7	Proteasome 20S subunit alpha 7
PSMB1	Proteasome 20S subunit beta 1
PSMD4	Proteasome 26S subunit ubiquitin receptor, non–ATPase 4
PSMD11	Proteasome 26S subunit, non–ATPase 11
PSMB4	Proteasome 20S subunit beta 4
PSMD9	Proteasome 26S subunit, non–ATPase 9
NFKBIA	NFKB inhibitor alpha

### Identification of Immune-Related Differentially Expressed Genes in SCM

**Figure 6:**
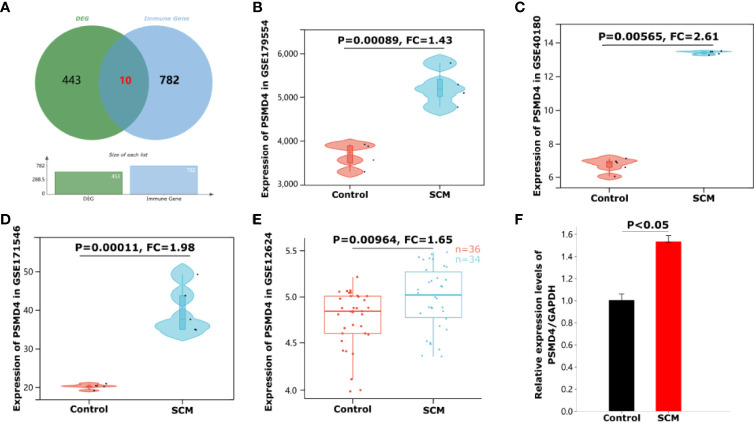
Identification and validation of immune-related hub genes PSMD4 in SCM. **(A)** Venn diagram showing 10 intersection genes of differential genes and immune cell marker genes. **(B)** The violin plot represents the expression of PSMD4 in mice cardiomyocytes in the dataset GSE179554. **(C)** The violin plot represents the expression of PSMD4 in mice cardiomyocytes in the dataset GSE40180. **(D)** The violin plot represents the expression of PSMD4 in mice cardiomyocytes in the dataset GSE171546. **(E)** The box plot represents the expression of PSMD4 in human peripheral whole blood in the dataset GSE12624. **(F)** Detection of PSMD4 expression using qRT-PCR. The expression of PSMD4 was significantly upregulated.

We intersected 453 shared DEGs with 782 IRGs and obtained 10 immune genes present in SCMs: STX16, CEP68, SMAD1, ZBTB16, PSMD4, HSD11B1, PPARG, SLC7A8, THBS1, and ASPSCR1 (**Figure 6A**). Details of the 10 IRGs are proven in **Table 3**. This suggests that these IRGs play crucial roles in the immune response of SCM. Because SCM is an acute aggravating ailment induced *via* circulatory dysfunction and immune gadget imbalance brought on by way of infection, the steady-state rules and protection feature of the immune machine significantly have an effect on the prognosis of SCM. To locate the key targets of SCM, we intersected 10 immune genes and 10 hub genes of cytoHubba. Finally, we obtained an IRHG-PSMD4, which was upregulated in SCM (FC = 1.43, *P* = 0.00089 in GSE179554; FC = 2.61, *P* = 0.00565 in GSE40180) (**Figures 6B, C**). To additionally verify whether or not PSMD4 is a normal gene in SCM ailment states, we validated PSMD4 with the GSE171546 and GSE12624 datasets. The GSE171546 dataset has a total of 20 samples, and we used five normal myocardial tissue data and five CLP-induced sepsis mouse model myocardial tissue data to verify the expression of PSMD4. GSE12624 collected 70 human samples, including 36 control samples and 34 SCM samples. The results showed that PSMD4 had a trend consistent with previous results, being upregulated and statistically significant in both the mouse dataset GSE171546 (FC = 1.98, *P* = 0.00011) (**Figure 6D**) and the human dataset GSE12624 (FC = 1.65, *P* = 0.00964) (**Figure 6E**).

**Table 3:** Top 10 genes that intersect with immune genes among differential genes.

Gene symbol	Description
STX16	Syntaxin 16
CEP68	Centrosomal protein 68
SMAD1	SMAD family member 1
ZBTB16	Zinc finger and BTB domain containing 16
PSMD4	Proteasome 26S subunit ubiquitin receptor, non–ATPase 4
HSD11B1	Hydroxysteroid 11-beta dehydrogenase 1
PPARG	Peroxisome proliferator activated receptor gamma
SLC7A8	Solute carrier family 7 member 8
THBS1	Thrombospondin 1
ASPSCR1	ASPSCR1 tether for SLC2A4, UBX domain containing

### Validation of Immune-Related Hub Genes

We assessed the expression of the IRHG-PSMD4. qRT-PCR was used to detect the gene expression of PBMCs in three patients with SCM and three normal people. The results of qRT-PCR had a consistent trend with the analysis results: as shown in **Figure 6F**, the expression level of PSMD4 in PBMCs of patients with SCM was significantly higher than that of normal controls (*P* < 0.05).

### Prediction of Target miRNAs and Construction of the Co-Expressed Network

**Figure 7:**
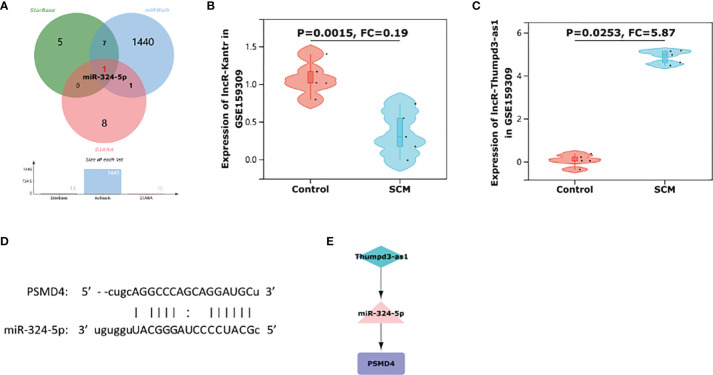
Construction of immune-related ceRNA network Thumpd3-as1/miR-324-5p/PSMD4. **(A)** A Venn diagram of shared target miRNAs of PSMD4 of the three databases. **(B)** The violin plot represents the expression of Kantr in rat cardiomyocytes in the dataset GSE159309. **(C)** The violin plot represents the expression of Thumpd3-as1 in rat cardiomyocytes in the dataset GSE159309. **(D)** Binding site map of miR-324-5p and PSMD4. **(E)** Thumpd3-as1/miR-324-5p/PSMD4 ceRNA network.

We used three online miRNA databases: starBase, miRWalk, and DIANA-TarBase, to predict the target miRNAs of PSMD4. The miRNA prediction results of PSMD4 are shown in **Supplementary Table S1**. The miRNA prediction results showed that miR-324-5p coexisted in the three databases (**Figure 7A**), suggesting that miR-324-5p is an effective miRNA of PSMD4. Thus, the co-expressed network of PSMD4 and target miRNAs was constructed: miR-324-5p/PSMD4.

### Construction of Immune-Related lncRNA–miRNA–IRHG Regulatory Network

**Figure 8:**
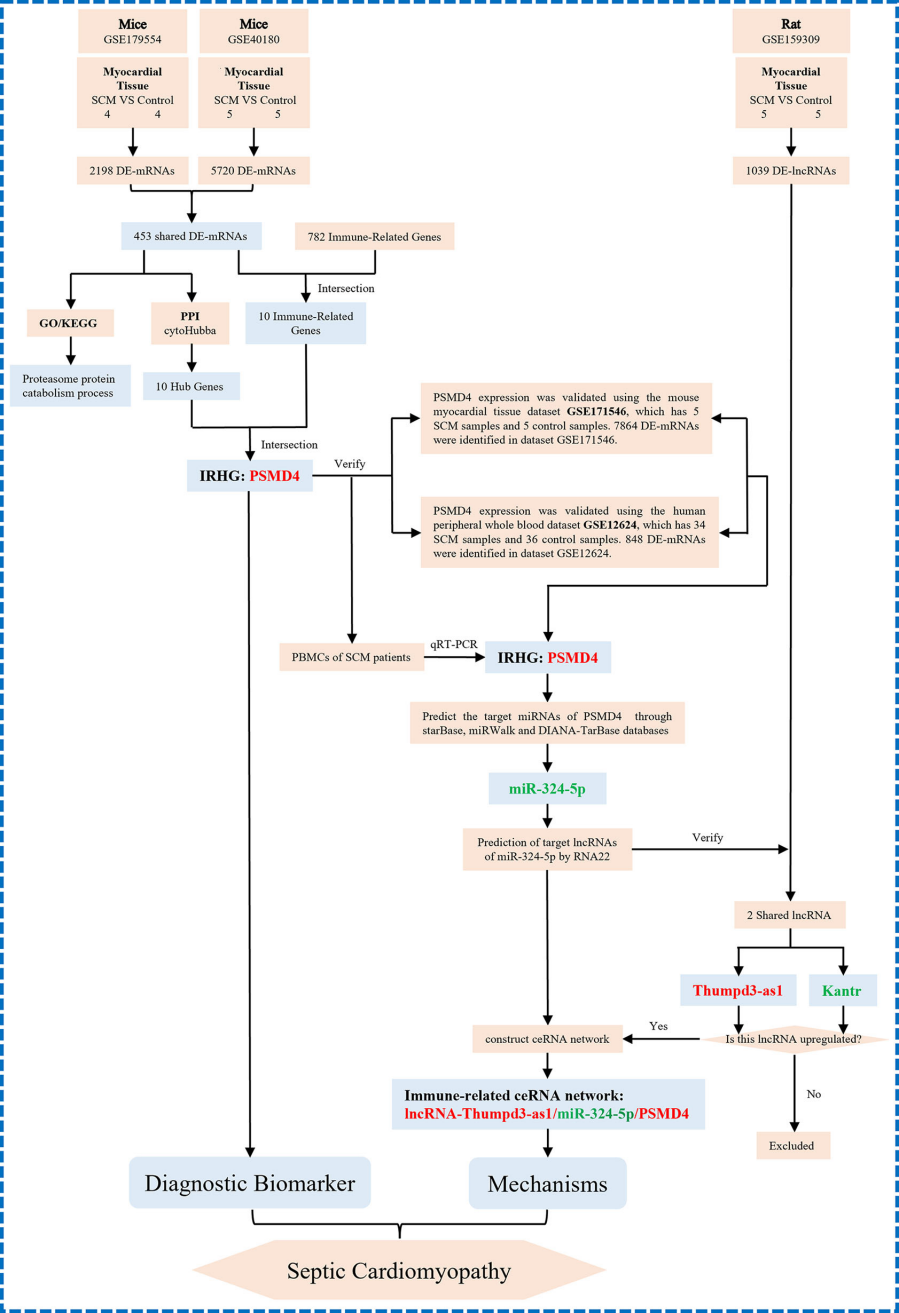
The technical workflow of this article and we proposed the novel ceRNA network. The red word means upregulation, and the green word means downregulation. IRHGs, immune-related hub genes.

miRNAs are well known to induce gene silencing and downregulate gene expression by binding mRNAs. However, its upstream molecules, lncRNAs, can affect the function of miRNA by combining MREs, thus upregulating gene expression. This interaction between RNAs is called a ceRNA network (Salmena et al., 2011). Next, we used the online database RNA22 to predict lncRNAs that interact with miR-324-5p. The prediction results of lncRNAs are shown in **Supplementary Table S2**. Through database prediction, we obtained 2,955 lncRNAs with potential binding sites for miR-324-5p. We then validated the lncRNAs using dataset GSE159309, which includes five lipopolysaccharide (LPS)–induced sepsis rat myocardial tissue data and five normal myocardial tissue data. Finally, after validation on the GSE159309 dataset, we obtained two lncRNAs for miR-324-5p from 2,955 predicted lncRNAs. One lncRNA (Kantr, FC = 0.19, *P* = 0.0015) (**Figure 7B**) was downregulated in SCM and one lncRNA (Thumpd3-as1, FC = 5.87, *P* = 0.0253) (**Figure 7C**) was upregulated in SCM. On the basis of the ceRNA hypothesis, we selected lncRNAs upregulated in SCM from these validated lncRNAs for ceRNA network construction. The binding sites of miR-324-5p and PSMD4 are shown in **Figure 7D**. Finally, an immune-related Thumpd3-as1/miR-324-5p/PSMD4 ceRNA network involved in the regulation of SCM mechanisms was constructed (**Figure 7E**), and the workflow of our study is summarized in **Figure 8**.

## Discussion

In the 1980s, Parker et al. evaluated cardiac characteristics in patients with septic shock and found that patients with septic cardiomyopathy had reduced LVEF and organic left ventricular enlargement (Parker et al., 1984). The high mortality rate of septic cardiomyopathy is due to the fact that it is a multi-organ–related infectious disease that is harmful to the whole body, resulting in dysfunction of the immune system and multiple organs (Hollenberg and Singer, 2021). At the same time, the activation of the body’s inflammatory response further impairs cardiomyocyte function (Brown and Jones, 2004). Therefore, the search for specific and highly sensitive diagnostic biomarkers is crucial for the early intervention of SCM. In recent years, target gene therapy has achieved remarkable results in the early diagnosis, treatment, and prognosis prediction of the disease (Dmitrieva et al., 2020; Sharma et al., 2020), enlightening that we can explore the early diagnosis and treatment biomarkers of SCM at the gene level.

Immune response is an important pathological process of SCM and participates in the regulation of SCM process (Zhang et al., 2020). However, the roles of IRGs in the development of SCM are currently unclear. Therefore, we combined shared DEG profiles in septic cardiomyopathy with IRGs to obtain IRHGs and immune-related ceRNA regulatory networks in SCM to deepen our understanding of the molecular mechanisms of septic cardiomyopathy.

After the construction of the PPI network, we identified 10 differentially expressed hub mRNA (PSMC4, PSMD7, PSMA5, PSMA7, PSMB1, PSMD4, PSMD11, PSMB4, PSMD9, and NFKBIA) using cytoHubba. These hub genes may be potential targets for the regulation in SCM. After verification of IRGs, PSMD4 was finally identified as IRHGs. PSMD4 (also known as Rpn10, offical full name: proteasome 26S subunit ubiquitin receptor, non–ATPase 4) is a subunit of regulatory granules, is the main ubiquitin (Ub) receptor for the 26S proteasome. Our results showed that PSMD4 is not only a central gene but also an IRG in SCM. At present, there are several studies showed that PSMD4 could affected the development of most tumor disease. For example, the study of Jiang et al. showed that PSMD4 can regulate the level of hypoxia-inducible factor 1α through the PTEN/Akt signaling pathway, thereby promoting the occurrence of hepatocellular carcinoma (Jiang et al., 2019); PSMD4 can inhibit endoplasmic reticulum (ER) stress–induced cells apoptosis, advertising the development of esophageal cancer (Ma et al., 2019); in addition, Xu et al. (2021) showed that PSMD4 is a potential target in mouse colorectal cancer, mediating the enhancement of immunoproteasome activity by atractylenolide I (ATT-I), making the body more responsive to immune checkpoint blocking therapy. However, studies on PSMD4 in septic cardiomyopathy are still insufficient. Therefore, we believe that PSMD4 holds promise as a novel immune-related biomarker and therapeutic target in SCM.

In addition, although immune responses are known to be an important pathological process in SCM, it is uncommon to construct immune-related ceRNA networks in SCM. Here, we constructed a ceRNA network targeting PSMD4 to explore the potential regulation of PSMD4 in SCM immunity. In this study, Thumpd3-as1/miR-324-5p/PSMD4 signaling pathway was identified by bioinformatic analysis.

### Thumpd3-as1/miR-324-5p/PSMD4 Axis

After confirming that PSMD4 is the hub gene of SCM, we estimated its target miRNA and finally got miR-324-5p. Because the main way that miRNA regulates target genes is to inhibit the expression of mRNA or promote the degradation of mRNA (Correia de Sousa et al., 2019), that is, miRNA is negatively correlated with mRNA expression. Our results showed that the IRG-PSMD4 was upregulated in SCM. Therefore, PSMD4 upstream miRNAs should be at low levels. Wang et al. found that the level of miR-324-5p in hypoxic/reoxygenated cardiomyocytes decreased with prolonged hypoxia, and miR-324-5p altered mitochondrial morphology and cardiomyocyte death by acting on Mtfr1 (Wang et al., 2015); the results in the work of Chen et al. confirmed that the expression level of miR-324-5p in peripheral blood endothelial progenitor cells of patients with ST-segment elevation myocardial infarction (STEMI) was significantly lower than that of healthy volunteers (Chen et al., 2019). In addition, Helwak et al. confirmed that miR-324-5p and PSMD4 have direct binding sites by CLASH technique (Helwak et al., 2013). These studies all demonstrate that it is realistic to construct a miR-324-5p/PSMD4 regulatory pathway. However, the specific mechanism of miR-324-5p/PSMD4 in SCM has not been reported yet, and further studies are needed.

Then, we further explored the lncRNAs that bind to miR-324-5p. We obtained two consensus lncRNAs (Kantr, FC = 0.19, *P* = 0.0015; and Thumpd3-as1, FC = 5.87, *P* = 0.0253) by RNA22 database prediction and GSE159309 dataset validation. Finally, Thumpd3-as1, a lncRNA with high expression in SCM, can bind to miR-324-5p according to the ceRNA network hypothesis (Smillie et al., 2018).

Previous research on Thumpd3-as1 (THUMPD3 antisense RNA 1) is mainly in oncology and osteoarthritis. Hu et al. confirmed that Thumpd3-as1 can regulate the self-renewal of non–small cell lung cancer cells (Hu et al., 2019); the findings of Wang et al. confirmed that Thumpd3-as1 overexpression enhanced the inflammatory response of chondrocytes in osteoarthritis (Wang et al., 2021); the study of Xia et al. confirmed that Thumpd3-as1 is a lncRNA related to the immune microenvironment of colon adenocarcinoma (Xia et al., 2021). All of these suggest that Thumpd3-as1 has strong feasibility in cellular characterization and immune regulation. However, the specific effects of Thumpd3-as1 and Thumpd3-as1/miR-324-5p/PSMD4 ceRNA network in the mechanism of SCM still need to be further explored.

As we pointed out in **Table 4**, two previous papers reported that identification of hub genes in human septic cardiomyopathy using bioinformatic analysis (Chen et al., 2020; Kang et al., 2020). Here, we presented that several findings in our study are significantly novel in comparing to what these two published papers. First of all, we used two datasets for DEGs acquisition and dataset validation for targets, which was solid. Furthermore, we identified IRHGs rather than common hub gene in SCM; in addition, a novel immune-related ceRNA network, Thumpd3-as1/miR-324-5p/PSMD4, was constructed and validated, which was different from the previous papers.

**Table 4:** Several findings in this study are significantly novel in comparing to what is published.

Items	Identification of Hub Genes in SCM Based on Bioinfomatics Analysis		
	Our Findings	Cell Paper (PMID:31424269)	Cell Paper (PMID:31794266)
Years	2022	2019	2020
Test set	GSE401807 and GSE179554	GSE79962	GSE79962
Species/tissue	Mice/Heart	Human/Heart	Human/Heart
Hub genes	IRHG: PSMD4	MYC, SERPINE1, CCL2 STAT3	NDUFB5, TIMMDC1, VDAC3
Validation set	GSE171546 and GSE159309	–	–
Verification	Human PBMC: qRT-PCR	–	Mice heart: WB
Mechanism	lncRNA–miRNA–IRHG ceRNA network: Thumpd3-as1/miR-324-5P/PSMD4	TF-miRNA-mRNA network	–

WB, Western blot.

Above all, this study explored biomarker genes in SCM using multiple GEO datasets of septic cardiomyopathy mouse heart tissue. In addition, we innovatively combined the hub gene of septic cardiomyopathy with the immune gene, obtained the important immune hub gene PSMD4 in SCM, and constructed the immune-related Thumpd3-as1/miR-324-5p/PSMD4 ceRNA network. It is suggested that PSMD4 and Thumpd3-as1/miR-324-5p/PSMD4 ceRNA network may be important biomarkers and potential therapeutic targets in SCM.

However, there are still some limitations of this study that need to be addressed. First, in our study, datasets of human and animal SCM model samples were used for analysis, but the sample size of peripheral blood from patients with SCM used for evaluation and validation in this study was small. Further increasing the sample size and conducting a potential cohort study to demonstrate our findings is what we need to do in the future. Second, although human samples were used for validation in this study, we used only peripheral blood single nucleated cells from patients with SCM for analysis due to the difficulty in obtaining live myocardial tissue. Obtaining cardiac tissues from patients with SCM and performing gene expression analysis under possible future conditions would improve the credibility and authenticity of the results of this study. Last, the association of immune genes with disease and disease severity requires further estimation due to lack of data including disease phenotype and disease activity status.

## Data Availability Statement

Publicly available datasets were analyzed in this study. This data can be found at https://www.ncbi.nlm.nih.gov/geo/query/acc.cgi GSE179554, GSE40180, GSE171546, SE159309 and GSE12624.

## Author Contributions

All authors have made significant contributions to the design and conception of the study, as well as data acquisition, analysis, and interpretation, and participated in the drafting or revision of the manuscript. For the final version to be published, all authors have made the final approval, and have reached an agreement with the journal to which the article is submitted, and are willing to be responsible for all aspects of the work to ensure the accuracy or completeness of any part of the article. All the authors are willing to cooperate in the investigation and resolution of related issues in the follow-up of the article. All authors contributed to the article and approved the submitted version.

## Funding

This study was supported by the National Natural Science Foundation of China (No. 81860073 and 81760074), Special Foundation Projects of Joint Applied Basic Research of Yunnan Provincial Department of Science and Technology with Kunming Medical University [No. 2019FE001(-138)], Yunnan Provincial Department of Science and Technology (No. 202001AT070039), Yunnan Health Training Project of High Level Talents (No. H-2018032), 100 Young and Middle-aged Academic and Technical Backbones of Kunming Medical University (No. 60118260106), Young Talents of Yunnan Thousand Talents Plan (RLQN20200002), and Clinical Medcial Center for Cardiovascular and Cerebrovascular Disease of Yunnan Province (No. ZX2019-03-01).

## Conflict of Interest

The authors declare that the research was conducted in the absence of any commercial or financial relationships that could be construed as a potential conflict of interest.

## Publisher’s Note

All claims expressed in this article are solely those of the authors and do not necessarily represent those of their affiliated organizations, or those of the publisher, the editors and the reviewers. Any product that may be evaluated in this article, or claim that may be made by its manufacturer, is not guaranteed or endorsed by the publisher.
